# ASD v2.0: updated content and novel features focusing on allosteric regulation

**DOI:** 10.1093/nar/gkt1247

**Published:** 2013-11-28

**Authors:** Zhimin Huang, Linkai Mou, Qiancheng Shen, Shaoyong Lu, Chuangang Li, Xinyi Liu, Guanqiao Wang, Shuai Li, Lv Geng, Yaqin Liu, Jiawei Wu, Guoqiang Chen, Jian Zhang

**Affiliations:** ^1^Department of Pathophysiology, Chemical Biology Division of Shanghai Universities E-Institutes, Key Laboratory of Cell Differentiation and Apoptosis of Chinese Ministry of Education, Shanghai Jiao-Tong University School of Medicine (SJTU-SM), 280 Chongqing Road, Shanghai 200025, China, ^2^Medicinal Bioinformatics Center, Shanghai Jiao-Tong University School of Medicine (SJTU-SM), 280 Chongqing Road, Shanghai 200025, China and ^3^Department of Urology, The Second Affiliated Hospital of Dalian Medical University, 467 Zhongshan Road, Dalian, 116023, China

## Abstract

Allostery is the most direct and efficient way for regulation of biological macromolecule function and is induced by the binding of a ligand at an allosteric site topographically distinct from the orthosteric site. AlloSteric Database (ASD, http://mdl.shsmu.edu.cn/ASD) has been developed to provide comprehensive information on allostery. Owing to the inherent high receptor selectivity and lower target-based toxicity, allosteric regulation is expected to assume a more prominent role in drug discovery and bioengineering, leading to the rapid growth of allosteric findings. In this updated version, ASD v2.0 has expanded to 1286 allosteric proteins, 565 allosteric diseases and 22 008 allosteric modulators. A total of 907 allosteric site-modulator structural complexes and >200 structural pairs of orthosteric/allosteric sites in the allosteric proteins were constructed for researchers to develop allosteric site and pathway tools in response to community demands. Up-to-date allosteric pathways were manually curated in the updated version. In addition, both the front-end and the back-end of ASD have been redesigned and enhanced to allow more efficient access. Taken together, these updates are useful for facilitating the investigation of allosteric mechanisms, allosteric target identification and allosteric drug discovery.

## INTRODUCTION

Allostery is a fundamental process that regulates a protein’s functional activity through the induction of changes in its conformation and dynamics in response to the perturbation of an effector at a site distinct from the active site, also termed the allosteric site (1). The mechanisms of allosteric perturbation in cells are diverse, ranging from binding scenarios (with ions, lipids, small molecules, etc.) (2–5). The propagation of a perturbation signal across a protein’s 3D structure is an inextricable link to allosteric communication or pathways. In essence, allosteric regulation is an efficient mechanism that controls protein activity in most biological processes, encompassing signal transduction, metabolism, catalysis and gene regulation (6,7). Deregulation of the fine-tuned network of allosteric regulation encoded in proteins has been intimately implicated in the pathogenesis of human diseases, such as cancers, diabetes, inflammation and Alzheimer’s disease (8–10).

The universality and efficiency of allosteric regulation have drawn increasing attention as a potential novel mechanism for new drug development (11–13). Allosteric drugs have several important advantages over orthosteric drugs when targeting the same macromolecule, including quiescence in the absence of endogenous orthosteric activity, greater selectivity as a result of higher structural divergence in the allosteric site, and limited positive or negative cooperation imposing a ‘ceiling’ on the magnitude of the allosteric effect (14,15).

In 2010, we released AlloSteric Database (ASD v1.0) (16), the first database that provides a comprehensive allosteric resource to describe the specific structure, function and mechanism of 336 allosteric proteins and 8095 allosteric modulators. However, in the past 3 years, advances in understanding allostery and the widespread applications of biophysical methods, such as X-ray crystallography (17), solid-state and relaxation dispersion nuclear magnetic resonance (NMR) (18), H/D exchange mass spectrometry (19), high-throughput screening (17), patch-clamp fluorometry (20) and electrophysiology (21) create plenty of opportunities to recognize novel allosteric molecules, leading to an explosive growth in the number of allosteric proteins and allosteric modulators. Intrinsically, the allosteric regulation of proteins is triggered by the binding of a modulator to their allosteric sites; thus, it is useful to understand the knowledge of known allosteric sites involved in the allosteric regulation as well as the interactions between allosteric sites and modulators on a wider scale (14,15). Over the years, the allosteric mechanisms regulated by modulators in multimeric proteins have been well documented from the Monod-Wyman-Changeux (MWC) model (22), the Koshland-Nemethy-Filmer (KNF) model (23) to recent Morpheerin model (24–26). In addition to these concerted/sequential models, allosteric pathways achieved by the propagation of a perturbation signal from the allosteric site to the orthosteric site were experimentally observed in some proteins (e.g. CREB binding protein), which not only provides insight into the allosteric mechanism in various biological processes but also contributes to the explanation of some disease-associated mutations away from the orthosteric site (27).

Since the release of ASD v1.0, feedback from users has led to many excellent suggestions on how to expand and enhance ASD offerings. Likewise, continuing advances in the field of allostery have led to a substantial expansion of the allosteric data. Here, we have updated this database. In the new version of ASD 2.0, the allosteric proteins and allosteric modulators deposited have been approximately trebled to 1286 and 22 008, the allosteric interactions and allosteric diseases increased to 23 099 and 565, respectively, and for the first time, 218 unique allosteric site structures and 48 allosteric pathways are included. In addition to the largely expanded data volume, the search engine and browser functions are enhanced, and a number of new cross-links to related databases have been added. The data architecture and content of the download files have been redesigned for users to facilitate the analysis of the allosteric information. Both biological and chemical researchers may benefit from the more resource-rich updated version of ASD.

## EXPANDED DATABASE CONTENTS

**Figure 1.**
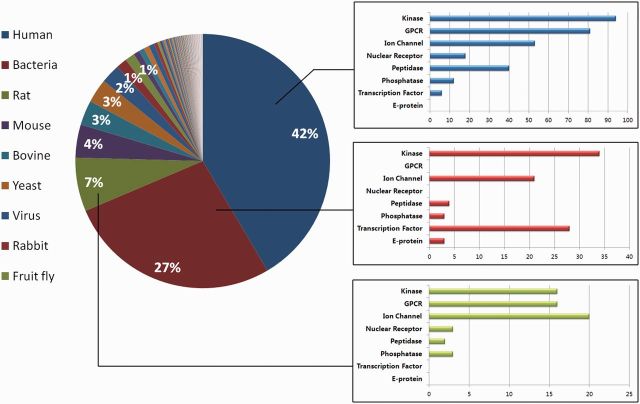
Species distribution of allosteric proteins in ASD v2.0. The number of allosteric proteins in eight target classes in the top three species is counted by histogram.

Additional allosteric proteins and chemical modulators, including structures, functions, related diseases and external links, were collected using the same methods described in our previous publication (16). In the current version, ASD contains all reported species instead of only the first reported species (in ASD v1.0) for each allosteric protein; 1286 allosteric proteins are deposited considering various species and 986 of them are non-redundant without considering species, as shown in Table 1. The number of allosteric proteins is dramatically augmented in the three categories classified in ASD v1.0, viz. kinases (from 46 to 190), GPCRs (from 48 to 109) and ion channels (from 21 to 119), which are primarily derived from exhaustive investigation of these key therapeutic targets by academia and pharmaceuticals industry in modern drug discovery (28–30). Meanwhile, several new classes of allosteric proteins such as peptidases, phosphatases, transcription factors and nuclear receptors have been gradually recognized for their important roles in the allosteric regulation during the recent years, and therefore 57 peptidases, 28 phosphatases, 46 transcription factors and 24 nuclear receptors were curated, annotated and deposited in ASD v2.0 (Table 1). The allosteric phenomenon has been widely observed in 181 different species, and the top three of species based on the number of allosteric proteins are human (42%), bacteria (27%) and rat (7%). As shown in Figure 1, allosteric kinases, GPCRs, and ion channels are most frequently found in humans and rats, whereas allosteric kinases, transcription factors and ion channels are most frequently found in bacteria. In addition, 565 diseases closely associated with the abnormality of the allosteric proteins were collected.

**Table 1.** Data statistics for allosteric proteins and modulators in updated ASD v2.0

Data category	ASD v2.0	ASD v1.0
Statistics of allosteric proteins^a^		
Number of all proteins	1286 (986)	336
Number of kinases	190 (131)	46
Number of GPCRs	109 (91)	48
Number of ion channels	119 (87)	21
Number of peptidases	57 (52)	0
Number of phosphatases	28 (19)	0
Number of transcription factors	46 (39)	0
Number of nuclear receptors	24 (21)	0
Number of E-proteins	5 (5)	2
Number of other proteins	708 (541)	219
Statistics of allosteric modulators		
Number of all modulators	22 008	8095
Number of activators	15 140	4784
Number of inhibitors	6207	3035
Number of regulators	850	386
Number of dual activators/regulators	47	16
Number of dual inhibitors/regulators	53	16
Number of dual activators/inhibitors	116	87
Number of multiple activators/ inhibitors/regulators	27	9
Statistics of allosteric protein interactions		
Number of protein-modulator interactions	23 099	8680
Statistics of allosteric protein structures		
Number of proteins with crystal/NMR structures	900	203
Number of protein-modulator crystal/NMR structures	907	156
Number of proteins with modeling structures	386	133
Statistics of allosteric diseases		
Number of allosteric related diseases	565	248

^a^The number of allosteric proteins in ASD v2.0 was counted as the sum of all reported species of each allosteric protein. The number in the brackets represents the sum of the first reported species for each allosteric protein, which was used in the statistics for allosteric proteins in ASD 1.0.

Currently, ASD 2.0 contains 22 008 allosteric chemical modulators composed of 15 140 activators, 6207 inhibitors and 850 regulators. The definition of the three classes was provided in our previous publication (16). Compared with the reported allosteric modulators by August 2010 (the release date of ASD v1.0), the number of experimentally determined activators, inhibitors and regulators in the past 3 years has increased by sharp increments of 3.16, 2.04 and 2.21 times. Among the modulators, 162 chemicals (∼0.7%), primarily drawn from endogenous allosteric chemical ligands, have >1 allosteric target with different allosteric effects, viz. dual activators/regulators, dual inhibitors/regulators, dual activators/inhibitors and multiple activators/inhibitors/regulators. For example, the endogenous theophylline can function as an allosteric activator for the adenosine receptor A1 (31) and as an allosteric inhibitor for the cAMP-specific 3′,5′-cyclic phosphodiesterase 4D (32); the endogenous ATP is involved in multiple allosteric regulation of targets, including cytosolic 5′-Nucleotidase II as an allosteric activator (33), glycogen phosphorylase α as an allosteric inhibitor (34) and aspartate carbamoyltransferase as an allosteric regulator (35). Due to the multiple targets of such modulators, there are 23 099 allosteric interactions between proteins and modulators recorded in ASD v2.0, larger than the total number of allosteric modulators.

To gain a clear insight into the relationship between allosteric function and structure, crystal/NMR structures of allosteric proteins in bound and unbound states have been manually curated from the PDB (36) since ASD v1.0. In the current release, the number of allosteric proteins with crystal/NMR structures has grown from 203 (ASD v1.0) to 900, covering up to 70.0% of all available allosteric proteins in our database. Moreover, 907 co-crystal/NMR structures were collected for 218 allosteric proteins bound to various allosteric modulators, increasing >5-fold in the past 3 years. For the remaining 30.0% of allosteric proteins without crystal/NMR structures, 386 modeling structures have been generated by the I-TASSER server (37) and included in ASD v2.0.

## NEW FEATURES

**Figure 2.**
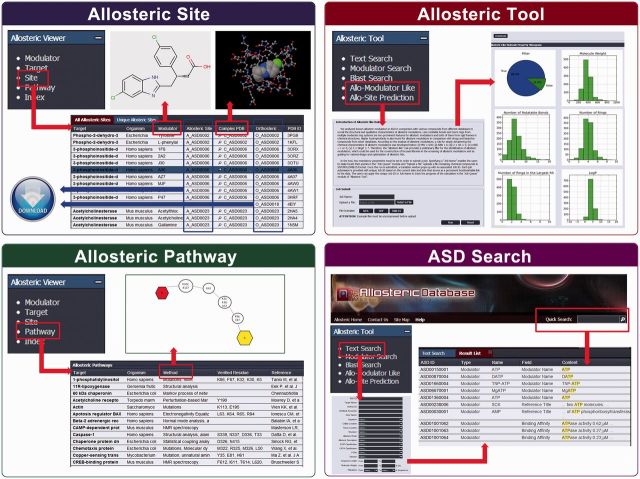
The new features and highlighted improvements in ASD v2.0.

Figure 2 presents and highlights new features of ASD v2.0, including the data integration of allosteric sites and the investigation of allosteric pathways. To facilitate the study of allosteric proteins and their modulators, the web interface and search engine have been redesigned and enhanced. Allosteric computational tools, original literature information related to allosteric entries and external cross-links with related databases are also provided in this online resource. The details of each new feature are depicted as follows.

### Allosteric sites

From the perspective of drug discovery, as allosteric sites evolved under lower sequence-conservation pressure compared with the evolutionarily conserved orthosteric sites, targeting allosteric sites can provide unprecedented advantages in terms of higher specificity, fewer side effects and lower toxicity (38). Understanding the characters of the available allosteric sites in proteins is a prerequisite for the identification of novel allosteric sites and the development of allosteric drugs. Therefore, the knowledge of allosteric sites is highly useful for facilitating allosteric discoveries and applications. In ASD v2.0, we have built a data set of the structures related to known allosteric sites. Of 23 099 experimental allosteric interactions, 907 allosteric protein-modulator complexes were determined by co-crystal/NMR structures. For each allosteric protein-modulator complex, three structural files in pdb format were constructed from the original PDB structure: the allosteric site, the allosteric site bound to the modulator and the corresponding orthosteric site (if available) of the protein. The residues constituting an allosteric site are automatically extracted from a complex structure by 6 Å around allosteric modulator in the site using Pymol (39) and manually inspected. If some residues of an orthosteric site are annotated in Pocketome (40), Catalytic Site Atlas (41) and BioLiP (42), all residues constituting the orthosteric site are detected by the fpocket algorithm (43) around the annotated residues and manually checked. These files can be accessed by clicking the ‘Site’ item under the menu of ‘Allosteric Viewer’ in the ASD home page, which opens the ‘All Allosteric Sites’ page. In the page, the target name and ID, the target species, the modulator name and a hyperlink to the original structure in PDB are also shown. Once a magnifier icon in the column of ‘Site Complex’ is clicked, a JMOL (http://www.jmol.org/) session will open. The session depicts the 3D structure of the allosteric site in stick representation and the modulator in sphere representation. The analysis of the key interactions from the allosteric site bound to a modulator may enhance the success rate for allosteric drug design, and the investigation of the structural pairs of allosteric sites and orthosteric sites could be useful in uncovering more potential allosteric pathways between them. A total of 907 allosteric site-modulator complexes contain 218 unique allosteric sites and 436 diverse chemical modulators. To highlight these known unique allosteric sites, these 218 allosteric sites with the best resolution of crystal structures are collected in the page of ‘Unique Allosteric Sites’ (Figure 2).

As shown in Table 2, the 218 known allosteric sites are mainly distributed within several classes of therapeutic targets, including kinases (19.3%), ion channels (5.0%) and transcription factors (4.1%). As one of the largest therapeutic targets, the known allosteric sites in GPCRs (1.8%) are rather limited, most likely due to the difficulty of crystallization (44), which severely impedes the progress of rational drug design based on the allosteric sites. With the recent technical breakthrough in GPCR crystallization, more allosteric sites of GPCRs are likely to be identified and located in the near future.

**Table 2.** Summary and statistics of newly added data in ASD v2.0

Data category	ASD 2.0
Statistics of allosteric sites^a^	
Number of all protein sites	218 (907)
Number of kinase sites	42 (121)
Number of GPCR sites	4 (4)
Number of ion channel sites	11 (47)
Number of peptidase sites	4 (9)
Number of phosphatase sites	4 (37)
Number of transcription factor sites	9 (58)
Number of nuclear receptor sites	7 (22)
Number of E-proteins sites	0 (0)
Number of other proteins sites	137 (609)
Statistics of allosteric pathways	
Number of all protein pathways	48
Number of kinase pathways	5
Number of GPCR pathways	3
Number of ions channel pathways	5
Number of peptidase pathways	2
Number of phosphatase pathways	1
Number of transcription factor pathways	3
Number of nuclear receptor pathways	2
Number of E-protein pathways	0
Number of other protein pathways	27

^a^The number of allosteric sites in ASD v2.0 was counted as the sum of the unique allosteric sites from crystal structures, and the number in the brackets represents the sum of allosteric modulators bound in the allosteric sites from crystal/NMR complex structures.

### Allosteric pathways

The increasing number of high-quality experimental data provides evidence that remote communication is a common phenomenon observed in the majority of proteins (45). A perturbation, such as modulator binding, creates a signal that propagates through non-covalent interactions from the allosteric site to the orthosteric site, which is the focus of allosteric pathway and is an important structural basis of the allosteric mechanism (13,26). Unraveling the potential allosteric pathways has increased the understanding of allosteric regulatory mechanisms and is used to guide a rational modulation of the physiopathological activities for therapy (6,45). In ASD v2.0, 48 allosteric pathways identified by experimental (e.g. site-directed mutagenesis, X-ray crystallography and NMR) and theoretical (e.g. Markov process, molecular dynamics simulation, geometrical interpretation and information theory) approaches (45–48) were collected from the published literature (Table 2), covering various classes such as kinases, ion channels, GPCR and so on. The allosteric pathway information can be accessed from the ‘Pathway’ item under the menu of ‘Allosteric Viewer’, including the target name and species, the method to detect the allosteric pathways, the experimentally verified residues in the allosteric pathway and the original references (Figure 2).

### Users interface improvements

To facilitate the use of ASD, both the front-end and the back-end of the web have been redesigned and enhanced to allow efficient access to the allosteric information of interest. First, a highly effective text search engine Sphinx (http://sphinxsearch.com/) is integrated into the back-end of ASD. The efficiency of text search was improved by ∼4-fold. On the front-end, a ‘Quick Search’ box has been added to the ASD home page, allowing users to quickly retrieve the information of interest by using Sphinx in ASD. An advanced search interface, ‘Text search’, under the ‘Allosteric Tool’ menu can be used to restrict the search by multiple options (Figure 2). Second, a high-performance chemical structure editor developed by our group, ChemV, is used for the ‘Modulator Search’ query. Compared with the java-based MarvinSketch in ASD v1.0, the loading of ChemV in the browser page is faster because no java plug-in is needed. Third, all front-end codes have been carefully revised to support most of major browsers, including Internet Explorer 7+, Firefox 3+, Chrome 9+, Safari 4+ and Opera 11+. Fourth, all the chemical structure information in ASD has been encoded into a central chemical structure database that allows more rapid querying, retrieval, monitoring and updating of structural data. Fifth, two allosteric computational tools developed by us are integrated under the ‘Allosteric Tool’ menu. ‘Allo-Modulator Like’ (49) provides a preliminary filter for the identification of potential allosteric modulators. ‘Allosite Prediction’ (50) is used to predict the allosteric sites of a protein. Finally, the XML file containing most of the ASD data has been redesigned to an easily parsed format. This redesign should make the development of data extraction routines much simpler and far faster for programmers and database developers.

## USE CASES

Most users will access the ASD database via its web interface to interactively query for allosteric information. Via the downloadable files under the ‘Download’ menu, ASD can also be used for large-scale computational studies that provide guidelines for experimental design. Using the allosteric protein data, Li *et al.* (51) and Namboodiri *et al.* (52) unraveled the physicochemical characters of allosteric proteins and allosteric sites. Huang *et al.* (50) and Panjkovich *et al.* (38) developed a computational approach to predict the position of allosteric sites in a protein, respectively. Based on the allosteric modulator data, Wang *et al.* (49) developed an empirical rule to determine the structural requirements for an allosteric modulator. ASD data has also been used to successfully design mutations in the allosteric site of fructose-1,6-bisphosphatase (FBPase) for rational protein engineering through evolutionary sequence analysis (53).

## CONCLUSION AND FUTURE DIRECTIONS

ASD is a comprehensive web-accessible allosteric database that brings together allosteric proteins, modulators and their associated experimentally confirmed data. Over the past 3 years, a significant expansion to the context and new features such as allosteric sites and allosteric pathways has been released in the current version of ASD. Additionally, the enhanced front-end and back-end of ASD now enable users to efficiently explore the available information about allostery. Overall, we believe these improvements should make ASD much more useful to the allosteric community for molecular mechanism studies, drug discovery and protein engineering. For the future development of ASD, in addition to regular updates of the knowledge-based content, we will focus on allosteric regulation in the cellular network, which is the composite of all interconnected pathways intersecting through shared allosteric macromolecules in the cell (54) and could be the origin of various syndromes and physiological abnormalities in the organism (28,55).

## FUNDING

National Basic Research Program of China (973 Program) [2011CB504001]; National Natural Science Foundation of China [81322046, 21002062, 21102090]; Shanghai Rising-Star Program [13QA1402300]; Program for Professor of Special Appointment (Eastern Scholar) at Shanghai Institutions of Higher Learning, the Program for New Century Excellent Talents in University [NCET-12-0355]; and the Graduate Student Innovation Fund of Shanghai Jiao Tong University School of Medicine [BXJ201202]. Funding for open access charge: National Basic Research Program of China (973 Program) [2011CB504001].

*Conflict of interest statement*. None declared.
